# Frequent Antenatal Care Visits Increase Institutional Delivery at Dabat Health and Demographic Surveillance System Site, Northwest Ethiopia

**DOI:** 10.1155/2019/1690986

**Published:** 2019-01-27

**Authors:** Abel Fekadu, Mezgebu Yitayal, Geta Asrade Alemayehu, Solomon Mekonnen Abebe, Tadesse Awoke Ayele, Amare Tariku, Gashaw Andargie, Destaw Fetene Teshome, Kassahun Alemu Gelaye

**Affiliations:** ^1^Department of Epidemiology and Biostatistics, Institute of Public Health, College of Medicine and Health Science, University of Gondar, Ethiopia; ^2^Department of Health Service Management and Health Economics, Institute of Public Health, College of Medicine and Health Science, University of Gondar, Ethiopia; ^3^Dabat Research Centre Health and Demographic Surveillance System, Institute of Public Health, College of Medicine and Health Science, University of Gondar, Ethiopia; ^4^Department of Human Nutrition, Institute of Public Health, College of Medicine and Health Science, University of Gondar, Ethiopia

## Abstract

**Background:**

Early diagnosis of pregnancy, professional follow-up, and skilled delivery service are the main interventions that reduce maternal morbidity and mortality. Generating local based evidence could support targeted and effective intervention placed by a government. Therefore, determining the prevalence of skilled institutional delivery and its associated factors is of supreme importance.

**Methods:**

A community based cross-sectional study was conducted among pregnant women at Dabat Health and Demographic Surveillance System (DHDSS) site from 2014 to 2015. A total of 1290 pregnant women were included in the study. Data were extracted from what was collected as part of the ongoing DHDSS. Variables were extracted from the Household Registration System (HRS2 version 2.1) database and exported to STATA version 14.1 for analysis. Binary logistic regression was used to identify the factors associated with skilled institutional delivery. Statistical test was considered significant at P value < 0.05.

**Results:**

The proportion of skilled institutional delivery was 31.0% (95% CI: 28.5, 33.6). Frequent Antenatal care (ANC) visits (Adjusted Odds Ratio (AOR): 2.94; 95% CI: 1.75, 4.94)), living in urban setting (AOR: 9.54; 95% CI: 5.99, 15.17), and ability to read and write (AOR: 1.81; 95% CI: 1.18, 2.75) were factors associated with increased delivery in the health institutions. On the other hand, giving more number of births (AOR: 0.39; 95% CI: 0.22, 0.66) decreased health institution delivery by 61%.

**Conclusion:**

Higher rate of skilled institutional delivery has been observed at the surveillance site as compared with the previous national estimates. Giving less number of births, frequent ANC visits, being in urban residence, and ability to read and write increased the likelihood of health institution delivery. Strengthening interventions that could influence the identified factors could improve mothers' choice to skilled institutional delivery.

## 1. Introduction

Globally, maternal mortality ratio (MMR) is estimated at 500/100,000 live births of which sub-Saharan countries constitute 56% [1, 2]. Ethiopia is one of the sub-Saharan African countries with the highest maternal mortality (420/100,000 live births) in the world, which is found to be far from the World Health Organization (WHO) target (267/100,000) for 2015 [3].

In sub-Saharan Africa, however, the high maternal and child mortality has been attributed to low coverage of maternal services during pregnancy, most of which is linked to extremely low utilization of skilled delivery services [4, 5]. According to the 2014 Ethiopian mini-Demographic and Health Survey (EDHS), 16% of women in the country gave births at health facilities; the figure was much lower (11.1%) in the Amhara Region [6]. Studies conducted in the country have reported different magnitudes of skilled delivery rate. For example, studies conducted in Arsi, Pastoralist Afar, Sekela district, northwest Ethiopia, Munisa woreda, southeast Ethiopia, and Wukro and Butajira Demographic and Health Surveillance sites reported 16.3% [7], 3.2% [8], 12.1% [9], 12.3% [10], and 25% [11] skilled institutional delivery, respectively.

Giving more number of births is known to be associated with less chance of choosing institutional delivery [12]. Educated mothers [13–15] and those who attended a minimum of one ANC service [14] were more likely to give birth at health institution than their counterparts. Wealth index was the other factor because mothers in the higher wealth were known to opt delivery at health institutions [16]. The age of the mother is also turned out to be the other factor associated with skilled delivery in the health institution as the service dropped when the age of the mother increases [17–20].

In Ethiopia, where only 16% of the births are delivered at health facilities by skilled health professionals, documenting the proportion and factors affecting the desire for such delivery services through a community based study is an important input for planning effective interventions. Additionally, the risk factors for institutional delivery are poorly documented and understood in northwest Ethiopia. The current study aimed at determining the prevalence of skilled delivery service utilization and its associated factors among DHDSS site mothers.

## 2. Methods

### 2.1. Study Area and Period

The study was conducted at DHDSS site from 2014 to 2015. Dabat is one of the 24 districts found in North Gondar zone, Amhara National Regional State, located about 820 km northwest of Addis Ababa. The site was established in 1996 in a total of 13 kebeles (the lowest administrative units in Ethiopia). Currently, the site includes 9 rural and 4 urban kebeles of the total 32 kebeles of the district with 17,000 households and 69,468 people.

The Dabat Health and Demographic Surveillance System is a full member of the International Network of Demographic Evaluation of Populations and Their Health (INDEPTH), a network of 44 DHSSs from the Global South. The detailed data collection system, data quality control, the database, and the study setting of the DHDSS site are described elsewhere.

### 2.2. Study Design and Population

This analysis was based on a community based longitudinal data collected from the DHDSS. We extracted and used the data collected as part of the DHDSS ongoing surveillance to determine an institutional skilled delivery service and to identify its associated factors in the years 2014 to 2015. We analyzed a data of 1290 pregnant women in the reproductive age group (15-49 years), who had a complete follow-up during their pregnancies and gave birth in the period between January 2014 and December 2015.

### 2.3. Data Collection Tools and Procedures

Dabat Health and Demographic Surveillance System site is a community surveillance which includes house-to-house visits that contribute to the ongoing and continuous population events registration of pregnancy (including outcome), ANC attendance, and place of delivery. Specially designed pregnancy and delivery (birth) update forms include variables that describe maternal sociodemographic and reproductive health characteristics.

Trained fieldworkers identified and registered all pregnancies, deliveries, and deaths during their routine household visits of the study area. To ensure a timely capture of all demographic events, field workers visit households where such events occur every month. These visits are performed in addition to the routine six monthly updates of all individuals in every household and location within the DHDSS catchment area. Each registered pregnancy is monitored until completion and then categorized as resulting in a live birth or stillbirth, an abortion, or outmigration if the pregnant woman moved out of the study area.

Skilled institutional delivery was considered when deliveries took place in health institutions and assisted by health professionals (doctors, health officers, nurses, midwives, or health assistants). Maternal residence, educational level, occupation, marital status, religion, and ethnicity were collected as sociodemographic variables. To characterize mothers by their obstetrics, we have included previous pregnancy outcomes, number of live births the mother ever had, and frequency of ANC visits the mother made [21].

### 2.4. Data Management and Analysis

The Dabat Health and Demographic Surveillance System site uses the Household Registration System (HRS version 2.1) database, which was customized from other DHSS sites in Africa.

Variables related to delivery service were extracted from the databases and exported to STATA version 14.1. Descriptive analysis, including proportions and cross tabs were made and presented using texts and tables. Variables that had a significant associations with institutional delivery (P value less than 0.2) in the bivariable binary logistic regression analysis were entered into a multivariable binary logistic regression model to identify the factors associated with skilled institutional delivery service utilization. Finally, variables that had a significant associations with institutional delivery service utilization were identified on the basis of their Adjusted Odds Ratio with a 95% CI and P value<0.05.

## 3. Results

### 3.1. Sociodemographic Characteristics of Pregnant Women

A total of 1290 pregnant women at the DHDSS site participated in the study. Of these, 1049 (81.32%) were married, 979 (75.89%) were housewives, 855 (66.28%) cannot read and write, and the majority (81.78%) lived in the rural parts of the district (Table 1).

### 3.2. Obstetric History of the Pregnant Women

Among the participants, 476 (36.91%) had four to eleven pregnancies, while three-fourths, 960 (74.42%), of the mothers had one live birth. Over half, 684 (53.02%), of the mothers made one to two visits for Tetanus Toxoid (TT) vaccine, while one-third, 430 (33.33%), made ≥4 ANC visits (Table 2).

### 3.3. Prevalence of Skilled Institutional Delivery

In this study, the prevalence of skilled institutional delivery was found to be 31.0% (95% CI: 28.53%, 33.59%). We found a high disparity of skilled delivery within the rural and urban settings (p <0.001).

### 3.4. Factors Associated with Skilled Institutional Delivery

After adjusting for sociodemographic and mothers obstetric characteristics; frequency of ANC visits, total number of pregnancies, residence, and educational status were significantly associated with skilled institutional delivery.

Accordingly, the odds of skilled institutional delivery among mothers who made ≥4 ANC visits to heath institutions were 2.94 times (AOR: 2.94; 95% CI: 1.75, 4.94) as likely as compared to mothers who had <4 ANC visits. Similarly, the odds of skilled institutional delivery decreased by 53% (AOR: 0.47; 95% CI: 0.28, 0.77) and 61% (AOR: 0.39; 95% CI: 0.22, 0.66) among mothers who had two to three and four to eleven pregnancies, respectively.

Urban dwellers were 9.54 times (AOR: 9.54; 95%CI: 5.99, 15.17) likely to have skilled delivery services in health institution compared to their rural counterparts. Educational status of a mother had a significant association with skilled institutional delivery. The odds of getting skilled delivery services at health institutions among mothers who read and write were 1.81 times (AOR: 1.81; 95% CI: 1.18, 2.75) likely compared to their counterparts (Table 3).

## 4. Discussion

Maternal mortality can be significantly reduced if all pregnant women get skilled delivery services at health institutions [22]. Nevertheless, the number of pregnant mothers who had skilled delivery services have not shown significant changes in the past decades [6, 23].

This study found out two-year skilled institutional deliveries of 31% among DHDSS site pregnant women, which is significantly higher than the mini-EDHS (16%) report [23]. Our finding was also significantly higher than those of studies in Arsi, Sekela district, northwest Ethiopia, Munisa woreda, southeast Ethiopia, and Wukro and Butajira DSS sites which reported 16.3% [7], 12.1% [9], 12.3% [10], and 25% [11], respectively. The difference might be attributed to the fact that during this period many interventions that might result in a better coverage were carried out by the government.

However, the current finding was lower than studies conducted in Sodo woreda, Bahir Dar town, and East Welega zone which reported 62.2% [17], 78.8% [24], and 39.7% [25], respectively. This discrepancy might account for real intervention differences among the study areas. On top of that, those study areas are exclusively urban where there is better access of health facilities and awareness of mothers about giving birth in health facilities.

This study also attempted to identify factors associated with skilled institutional delivery. Skilled institutional delivery and frequency of ANC visits had direct relationship; that is, the more times the mother went to health facilities, the better chance of getting skilled delivery service at health institutions. This finding is consistent with other studies conducted in different parts of the country [26–28]. It has been noted that one component of ANC is to provide information related to pregnancy outcomes and common planning of delivery options. This information could have helped mothers to choose health institutions to deliver their babies. Like other studies conducted in different parts of the country [18, 29], this work identified the associations between skilled institutional delivery and the number of pregnancies that the mother had. The odds of skilled institutional delivery was 53% and 61% lower among mothers who had two to three and four to eleven pregnancies compared to primigravida mothers. This could be attributed to the fact that the mother who had more number of pregnancies might undermine the risk and have more confidence in having healthy deliveries. On top of that, the existence of less risk and less occurrence of prolonged labor compared to primigravida mothers could affect their preference for health institution delivery.

The other variable which had a significant association with skilled institutional delivery service was mother's educational status. Accordingly, more educated mothers had more chance of choosing skilled institutional delivery service. Mothers who could read and write were 1.81 times likely to get skilled institutional delivery. The finding is similar to those of other studies conducted so far [9, 30]. It is expected that educated mothers have a high level of health awareness and greater knowledge about available health services; they are thus most likely to know and choose quality services and easily understand the risk behind home delivery.

Similarly, like other studies published on this area [20, 29, 30], this study identified the effect of women's residence on their institutional delivery choice. Urban women were 9 times as likely to get skilled institutional delivery service. This might be explained by the fact that urban mothers have better access to health facilities and information related to health institution delivery benefits.

As a limitation, unmeasured independent factors like economic status, knowledge of danger signs, attitude towards health professionals, husbands' approval and involvement, and quality of ANC and logistics of the health institutions might have important impacts on the outcomes that have not been captured in this study. Therefore, the application of this finding for community intervention and comparison should take the inherent limitation of the work into account.

## 5. Conclusion

The prevalence of skilled institutional delivery has shown a marked increase over previous EDHS research findings. Pregnant women who had lower number of births and frequent ANC visits, lived in urban areas, and were able to read and write were more likely to choose health institution delivery. Therefore, careful attention should be given to the increase in the literacy rate of rural mothers and to the promotion of the importance of frequent ANC visits.

## Figures and Tables

**Table 1:** Sociodemographic characteristics of pregnant mothers at DHDSS site, northwest Ethiopia, 2014-2015.

Characteristics	Number (Percent)
Ethnicity	
Amhara	1,288 (99.80)
Tigray	2 (0.20)
Religion	
Orthodox	1267 (98.22)
Muslim	23 (1.78)
Marital status	
Single	170 (13.18)
Married	1,049 (81.32)
Divorced	71 (5.50)
Educational status	
Not read and write	855 (66.28)
Read and write	435 (33.72)
Occupation	
All types of paid jobs	219 (16.98)
Housewife	979 (75.89)
Unemployed	92 (7.13)
Residence	
Rural	1,055 (81.78)
Urban	235 (18.22)

**Table 2:** Obstetric characteristics of pregnant mothers at DHDSS site, northwest Ethiopia, 2014-2015.

Characteristics	Number (Percent)
Total Number of pregnancies	
One (first pregnancy)	352 (27.28)
Two to three	462 (35.81)
Four to eleven	476 (36.91)
Parity (Birth)	
One	960 (74.42)
Two to three	234 (18.14)
Four to nine	96 (7.44)
TT vaccination history	
Not vaccinated	536 (41.55)
One to two times	684 (53.02)
Three to four times	70 (5.43)
Frequency of ANC visited	
Not attended	391 (30.31)
One to three times	469 (36.36)
Four and more times	430 (33.33)

**Table 3:** Bivariable and multivariable logistic regression analysis of factors associated with skilled institutional delivery at DHDSS site, northwest Ethiopia, 2014-2015.

**Variables**	**Delivery **		**COR, 95**%**CI**	**AOR,95**%**CI**
	**HI**	**Home**		
Frequency of ANC visited				
Not attended	105	286	1	1
One to three times	100	369	0.74 (0.54,1.01)	1.27 (0.77,2.12)
Four and more times	195	235	2.26 (1.68,3.03)	2.94 (1.75,4.94)*∗*
Total number of pregnancies				
First pregnancy	146	206	1	1
Two to three	162	300	0.77 (0.57,1.03)	0.47 (0.28,0.77)*∗*
Four to eleven	92	384	0.34 (0.25,0.82)	0.39 (0.22,0.66)*∗*
TT vaccination history				
Not vaccinated	174	362	1	1
One to two times	202	482	0.87 (0.64,1.08)	1.07 (0.71,1.63)
Three to four times	24	46	1.09 (0.62,1.91)	1.75 (0.85,3.61)
Residence				
Urban	195	40	20.21 (13.92,29.34)	9.54 (5.99,15.17)*∗*
Rural	205	850	1	1
Educational status				
Not read and write	157	698	1	1
Read and write	243	192	5.63 (4.35,7.27)	1.81 (1.18,2.75)*∗*
Occupation				
All types of paid jobs	131	88	5.05 (3.70,6.87)	1.58 (0.86,2.90)
Housewife	223	756	1	1
Job seeker	46	46	3.39 (2.19,5.24)	0.98 (0.47,2.05)
Mother's marital status				
Single	77	93	2.24 (1.61,3.12)	0.70 (0.37,1.72)
Married	283	766	1	1
Divorced	40	31	3.49 (2.14,5.69)	1.67 (0.81,3.46)

*∗*p value<0.05.

## Data Availability

The data used to support the findings of this study are available from the corresponding author upon request.

## Abbreviations

AOR: Adjusted Odds Ratio
ANC: Antenatal care
CI: Confidence interval
COR: Crude Odds Ratio
DHDSS: Dabat Health and Demographic Surveillance System
EDHS: Ethiopian Demographic and Health Survey
HI: Health institutions
HRS: Household Registration System
MDG: Millennium Development Goals
MMR: Maternal mortality ratio
SDG: Sustainable Development Goals
TT: Tetanus Toxoid
WHO: World Health Organization.
